# Deacetylation of α-tubulin and cortactin is required for HDAC6 to trigger ciliary disassembly

**DOI:** 10.1038/srep12917

**Published:** 2015-08-06

**Authors:** Jie Ran, Yunfan Yang, Dengwen Li, Min Liu, Jun Zhou

**Affiliations:** 1 State Key Laboratory of Medicinal Chemical Biology, College of Life Sciences, Nankai University, Tianjin 300071, China

## Abstract

Cilia play important roles in sensing extracellular signals and directing fluid flow. Ciliary dysfunction is associated with a variety of diseases known as ciliopathies. Histone deacetylase 6 (HDAC6) has recently emerged as a major driver of ciliary disassembly, but little is known about the downstream players. Here we provide the first evidence that HDAC6-mediated deacetylation of α-tubulin and cortactin is critical for its induction of ciliary disassembly. HDAC6 is localized in the cytoplasm and enriched at the centrosome and basal body. Overexpression of HDAC6 decreases the levels of acetylated α-tubulin and cortactin without affecting the expression or localization of known ciliary regulators. We also find that overexpression of α-tubulin or cortactin or their acetylation-deficient mutants enhances the ability of HDAC6 to induce ciliary disassembly. In addition, acetylation-mimicking mutants of α-tubulin and cortactin counteract HDAC6-induced ciliary disassembly. Furthermore, HDAC6 stimulates actin polymerization, and inhibition of actin polymerization abolishes the activity of HDAC6 to trigger ciliary disassembly. These findings provide mechanistic insight into the ciliary role of HDAC6 and underscore the importance of reversible acetylation in regulating ciliary homeostasis.

Cilia are antenna-like organelles protruding from the surfaces of most mammalian cells. Cilia are critically involved in many physiological and developmental processes, primarily due to their roles in sensing and transmitting environmental signals and orchestrating the flow of fluids^1^,^2^,^3^. Defects in ciliary structure and/or function are associated with a number of human diseases and developmental disorders, such as Bardet-Biedl syndrome, retinal degeneration and polycystic kidney, which are collectively called ciliopathies^1^,^2^,^3^. Both primary and motile cilia contain axonemes of nine outer microtubule doublets, which extend from the basal body and are surrounded by the ciliary membrane. A typical motile cilium also contains a pair of central microtubules and inner and outer dynein arms, which are important for ciliary motility and absent in the primary cilium^1^,^2^,^3^. Over the past decade, considerable progress has been made towards the characterization of ciliary composition and structure^4^, but the molecular mechanism for ciliary homeostasis is poorly understood.

Histone deacetylase 6 (HDAC6), a cytoplasmic member of the HDAC family, has recently been identified as an important driver of ciliary disassembly. For example, HDAC6 activation is required for Aurora A-dependent ciliary disassembly in the contexts of various signaling events mediated by proteins such as HEF1, MST1/2, calmodulin, inversin, β-catenin, and aPKC^5^,^6^,^7^,^8^,^9^,^10^,^11^,^12^. HDAC6 is also involved in epithelial ciliary dysfunction in response to cigarette smoke^13^. In addition, modulation of HDAC6 activity or localization has been shown to mediate the actions of CYLD, Dio3, Plk1, and ribosylation factor-like proteins in ciliogenesis^14^,^15^,^16^,^17^. However, the downstream players mediating the ciliary function of HDAC6 are largely unknown. A number of proteins, such as the microtubule component α-tubulin and the actin-binding protein cortactin, are known as HDAC6 substrates^18^,^19^,^20^,^21^,^22^,^23^,^24^, but it remains unclear whether the deacetylation of these proteins contributes to the ciliary role of HDAC6. In this study, we provide the first evidence that HDAC6-mediated ciliary disassembly is critically dependent on the deacetylation of both α-tubulin and cortactin.

## Results

### HDAC6 overexpression induces ciliary disassembly

**Figure 1:**
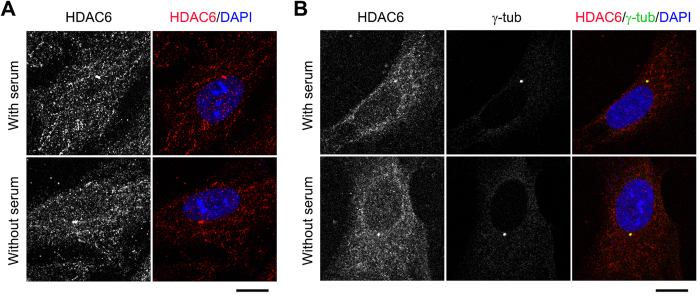
HDAC6 is enriched at the centrosome and basal body. (**A**) Immunofluorescence images of RPE1 cells cultured with or without serum for 24 hours and stained with HDAC6 antibody and DAPI. Scale bar, 5 μm. (**B**) Immunofluorescence images of RPE1 cells cultured with or without serum for 24 hours and stained with HDAC6 and γ-tubulin antibodies and DAPI. Scale bar, 5 μm.

**Figure 2:**
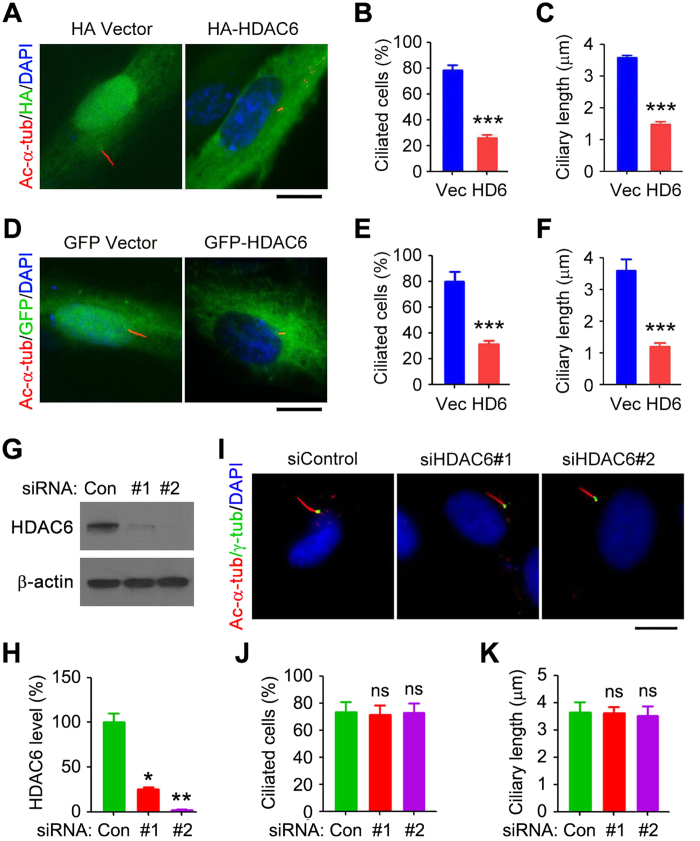
HDAC6 overexpression results in ciliary disassembly. (**A**–**C**) Immunofluorescence images (**A**), percentage of ciliated cells (**B**), and ciliary length (**C**) in RPE1 cells transfected with HA vector or HA-HDAC6, serum-starved for 24 hours, and stained with acetylated α-tubulin and HA antibodies and DAPI. Scale bar, 5 μm. (**D**–**F**) Immunofluorescence images (**D**), percentage of ciliated cells (**E**), and ciliary length (F) in RPE1 cells transfected with GFP vector or GFP-HDAC6, serum-starved for 24 hours, and stained with acetylated α-tubulin antibody and DAPI. Scale bar, 5 μm. (**G**) Western blot analysis of HDAC6 and β-actin in control or HDAC6 siRNA-treated RPE1 cells. (**H**) Experiments were performed as in G, and HDAC6 level was determined by densitometric analysis of the blots. (**I**–**K**) Immunofluorescence images (**I**), percentage of ciliated cells (**J**), and ciliary length (**K**) in RPE1 cells transfected with control or HDAC6 siRNAs, serum-starved for 24 hours, and stained with acetylated α-tubulin and γ-tubulin antibodies and DAPI. Scale bar, 5 μm. *P < 0.05, **P < 0.01, ***P < 0.001; ns, not significant. Error bars indicate SEM.

In this study, we analyzed the ciliary role of HDAC6 using hTERT-RPE1 cells (human telomerase-immortalized retinal pigment epithelial cells, hereinafter referred to as RPE1 cells), which are known to form primary cilia when cultured in the serum-free medium and have been widely used to investigate various aspects of cilia. Immunofluorescence staining with HDAC6 antibody or double staining with HDAC6 and γ-tubulin antibodies revealed that HDAC6 was localized in the cytoplasm and enriched at the centrosome and basal body (Fig. 1A,B). Both the percentage of ciliated cells and the length of cilia were significantly decreased by transfection with HA-HDAC6, compared with transfection with HA vector (Fig. 2A–C). Similar results were obtained by transfection of cells with GFP-HDAC6 (Fig. 2D–F). To further study the ciliary role of HDAC6, we depleted its expression by using two different siRNAs (Fig. 2G,H). Interestingly, loss of HDAC6 did not obviously affect the percentage of ciliated cells or the length of cilia (Fig. 2I–K). These results are in agreement with previous findings that the primary role of HDAC6 lies in ciliary disassembly^5^,^6^,^7^,^8^,^9^,^10^,^11^,^12^,^13^,^14^,^15^,^16^,^17^.

### The deacetylase activity of HDAC6 is important for its induction of ciliary disassembly

**Figure 3:**
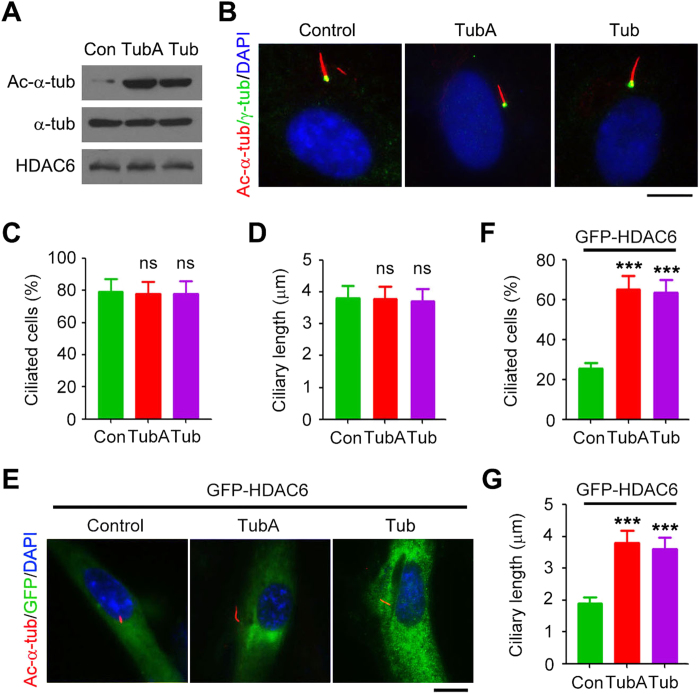
Pharmacological inhibition of HDAC6 activity protects cells from ciliary disassembly induced by HDAC6 overexpression. (**A**) Western blot analysis of acetylated α-tubulin, α-tubulin, and HDAC6 in RPE1 cells treated with tubastatin A (TubA, 2 μM), tubacin (Tub, 2 μM), or vehicle control for 4 hours. (**B**–**D**) Immunofluorescence images (**B**), percentage of ciliated cells (**C**), and ciliary length (**D**) in RPE1 cells treated with tubastatin A (2 μM), tubacin (2 μM), or vehicle control, serum-starved for 24 hours, and stained with acetylated α-tubulin and γ-tubulin antibodies and DAPI. Scale bar, 5 μm. (**E**–**G**) Immunofluorescence images (**E**), percentage of ciliated cells (**F**), and ciliary length (**G**) in RPE1 cells transfected with GFP-HDAC6, starved in serum-free medium containing tubastatin A (2 μM), tubacin (2 μM), or vehicle control for 24 hours, and stained with acetylated α-tubulin antibody and DAPI. Scale bar, 5 μM. ***P < 0.001; ns, not significant. Error bars indicate SEM.

To study whether HDAC6-induced ciliary disassembly requires its deacetylase activity, we treated RPE1 cells with tubastatin A and tubacin, two selective inhibitors of the deacetylase activity of HDAC6^25^,^26^. Both tubastatin A and tubacin efficiently inhibited HDAC6 activity, as indicated by the significant increase of α-tubulin acetylation (Fig. 3A). Tubastatin A and tubacin did not obviously affect the percentage of ciliated cells or ciliary length (Fig. 3B–D). However, both drugs could significantly protect cells from HDAC6-induced ciliary disassembly (Fig. 3E–G).

**Figure 4:**
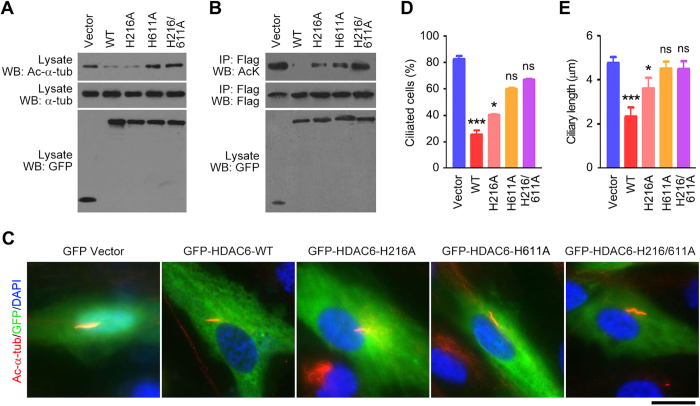
Mutation of the deacetylase domain of HDAC6 abolishes its activities to deacetylate α-tubulin and cortactin and to induce ciliary disassembly. (**A**) Western blot analysis of acetylated α-tubulin, α-tubulin, and GFP in RPE1 cells transfected with GFP vector or GFP-HDAC6 wild-type (WT), H216A, H611A, or H216/611A. (**B**) RPE1 cells were transfected with Flag-cortactin and GFP vector or GFP-HDAC6 WT, H216A, H611A, or H216/611A. The anti-Flag immunoprecipitate was then probed with acetylated lysine and Flag antibodies, and the cell lysate was probed with GFP antibody. (**C**–**E**) Immunofluorescence images (**C**), percentage of ciliated cells (**D**), and ciliary length (**E**) in RPE1 cells transfected with GFP vector or GFP-HDAC6 WT, H216A, H611A, or H216/611A, serum-starved for 24 hours, and stained with acetylated α-tubulin antibody and DAPI. Scale bar, 5 μm. *P < 0.05, ***P < 0.001; ns, not significant. Error bars indicate SEM.

To corroborate the above findings, RPE1 cells were transfected with various mutants of HDAC6. The mutants that we used include H216A (mutation of histidine 216 in the first deacetylase domain to alanine), H611A (mutation of histidine 611 in the second deacetylase domain to alanine), and H216/611A (mutation of both histidine 216 and histidine 611 to alanines). We found that H611A and H216/611A, but not H216A, remarkably abolished HDAC6-mediated deacetylation of α-tubulin in RPE1 cells (Fig. 4A). In addition, H216A and H611A partially inhibited HDAC6-mediated deacetylation of cortactin, whereas H216/611A entirely abolished the deacetylation of cortactin (Fig. 4B). We further found that while wild-type HDAC6 and H216A resulted in significant ciliary disassembly, H611A and H216/611A did not (Fig. 4C–E). Collectively, the above data suggest that the deacetylase activity is critical for HDAC6 to induce ciliary disassembly.

### HDAC6 overexpression does not affect the expression or localization of known ciliary regulators

**Figure 5:**
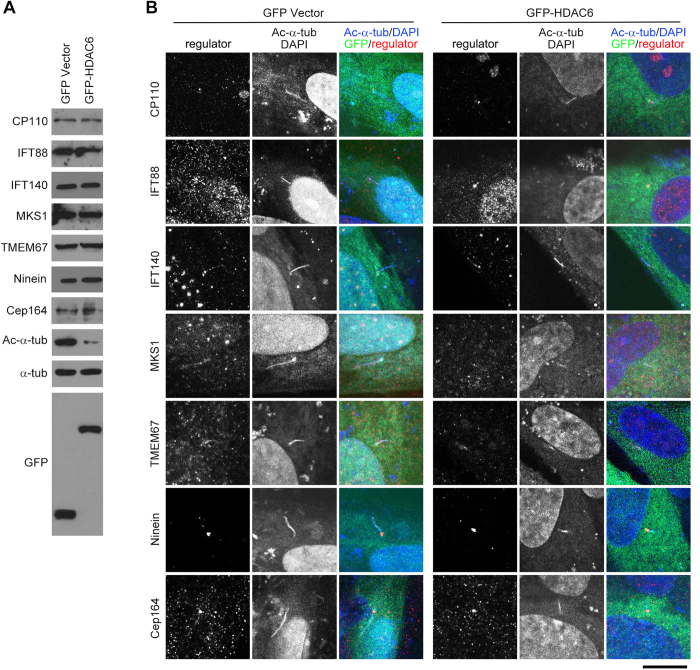
HDAC6 overexpression does not affect the expression or localization of known ciliary regulators. (**A**) Western blot analysis of the indicated ciliary regulators, acetylated α-tubulin, α-tubulin, and GFP in RPE1 cells transfected with GFP vector or GFP-HDAC6. (**B**) Immunofluorescence images of RPE1 cells transfected with GFP vector or GFP-HDAC6, serum-starved for 24 hours, and stained with antibodies against the indicated ciliary regulators and acetylated α-tubulin and DAPI. Scale bar, 5 μm.

We then sought to identify downstream proteins that mediate the ciliary role of HDAC6. We first analyzed the effect of HDAC6 overexpression on the expression of several key ciliary regulators, including centrosomal protein 110 (CP110), intraflagellar transport protein 88 (IFT88), IFT140, Meckel syndrome 1 (MKS1), ninein, and centrosomal protein 164 (Cep164)^27^,^28^,^29^,^30^,^31^. Western blot analysis revealed that transfection of RPE1 cells with GFP-HDAC6 did not obviously affect the expression of these ciliary regulators, although GFP-HDAC6 significantly decreased α-tubulin acetylation, compared with transfection with GFP vector (Fig. 5A). Immunofluorescence microscopy further showed that overexpression of HDAC6 did not alter the localization of these ciliary regulators, although it clearly resulted in the shortening of ciliary length (Fig. 5B). These results suggest that HDAC6 is likely to induce ciliary disassembly via mechanisms independent of these known ciliary regulators. We also found that siRNA-mediated depletion of HDAC6 expression did not affect the expression or localization of these ciliary regulators (Figure S1).

### Deacetylation of α-tubulin is required for HDAC6-mediated ciliary disassembly

**Figure 6:**
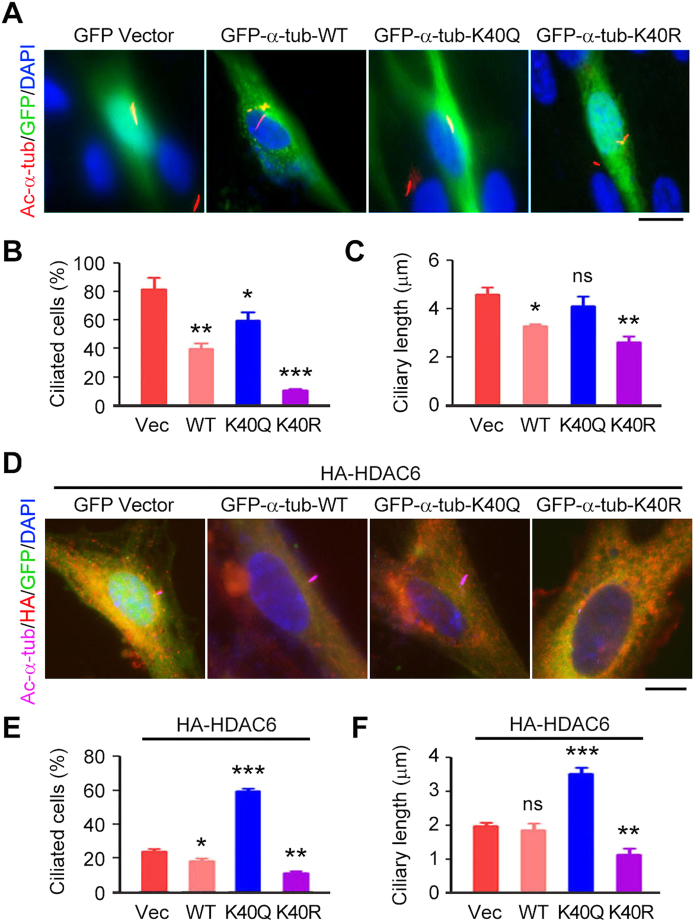
Deacetylation of α-tubulin is required for HDAC6-mediated ciliary disassembly. (**A–C**) Immunofluorescence images (**A**), percentage of ciliated cells (**B**), and ciliary length (**C**) in RPE1 cells transfected with GFP vector or GFP-α-tubulin wild-type (WT), K40Q, or K40R, serum-starved for 24 hours, and stained with acetylated α-tubulin antibody and DAPI. Scale bar, 5 μm. (**D**–**F**) Immunofluorescence images (**D**), percentage of ciliated cells (**E**), and ciliary length (**F**) in RPE1 cells transfected with HA-HDAC6 and GFP vector or GFP-α-tubulin WT, K40Q, or K40R, serum-starved for 24 hours, and stained with HA and acetylated α-tubulin antibodies and DAPI. Scale bar, 5 μm. *P < 0.05, **P < 0.01, ***P < 0.001; ns, not significant. Error bars indicate SEM.

To examine whether the level of α-tubulin acetylation is involved in ciliary homeostasis or is merely a ciliary marker, RPE1 cells were transfected with GFP-α-tubulin wild-type or mutants, including K40Q (mutation of lysine 40 to glutamine to mimic acetylation) and K40R (mutation of lysine 40 to arginine to disrupt acetylation). We found that transfection of RPE1 cells with GFP-α-tubulin wild-type or K40R significantly decreased the percentage of ciliated cells and the length of cilia, compared with transfection with GFP vector (Fig. 6A–C). By contrast, transfection with K40Q slightly decreased the percentage of ciliated cells and did not obviously affect ciliary length (Fig. 6A–C). Cotransfection of β-tubulin with α-tubulin resulted in the same phenotype as transfection of α-tubulin alone (Figure S2), ruling out the possibility that the expression of just α-tubulin causes a dominant effect on ciliary formation.

Since HDAC6 causes α-tubulin deacetylation, the next question then is whether the deacetylation of α-tubulin is required for HDAC6 to trigger ciliary disassembly. To answer this question, RPE1 cells were transfected with HA-HDAC6 together with GFP-α-tubulin wild-type, K40Q, or K40R. We found that α-tubulin wild-type and K40R could enhance the ability of HDAC6 to induce ciliary disassembly, whereas K40Q counteracted the ciliary effect of HDAC6 (Fig. 6D-F). These data suggest that deacetylation of α-tubulin contributes to HDAC6-mediated ciliary disassembly.

### Deacetylation of cortactin is required for HDAC6-mediated ciliary disassembly

**Figure 7:**
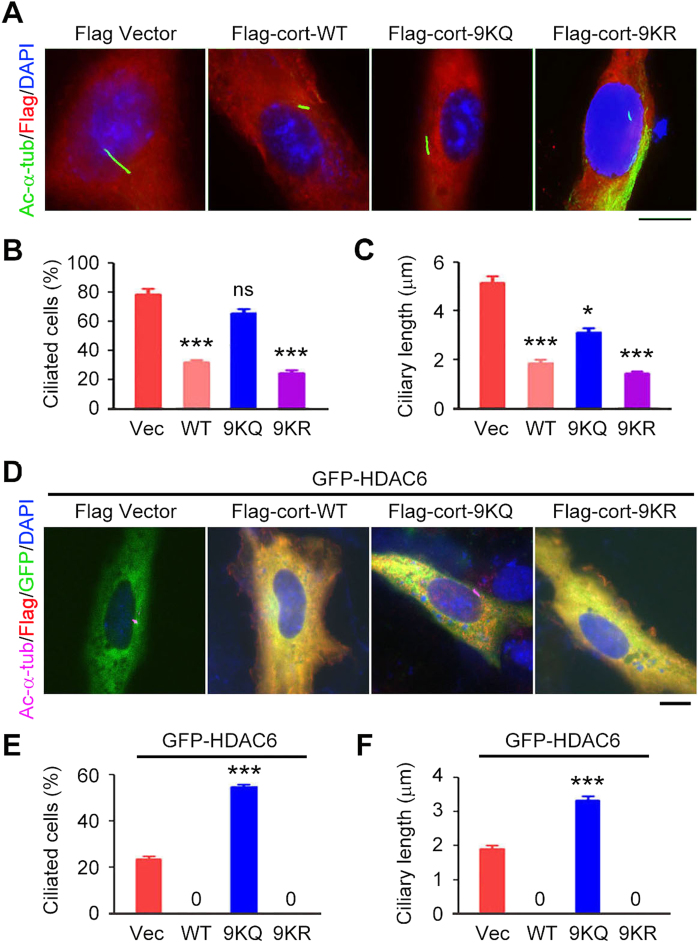
Deacetylation of cortactin is required for HDAC6-mediated ciliary disassembly. (**A**-**C**) Immunofluorescence images (**A**), percentage of ciliated cells (**B**), and ciliary length (**C**) in RPE1 cells transfected with Flag vector or Flag-cortactin wild-type (WT), 9KQ, or 9KR, serum-starved for 24 hours, and stained with acetylated α-tubulin and Flag antibodies and DAPI. Scale bar, 5 μm. (**D**-**F**) Immunofluorescence images (**D**), percentage of ciliated cells (**E**), and ciliary length (**F**) in RPE1 cells transfected with GFP-HDAC6 and Flag vector or Flag-cortactin WT, 9KQ, or 9KR, serum-starved for 24 hours, and stained with acetylated α-tubulin and Flag antibodies and DAPI. Scale bar, 5 μm. *P < 0.05, ***P < 0.001; ns, not significant. Error bars indicate SEM.

Cortactin is known to interact with filamentous actin (F-actin) and promote actin polymerization^32^,^33^,^34^. In addition, the acetylation of cortactin is known to impede its interaction with F-actin^20^. However, it is unknown whether the deacetylation of cortactin is involved in the action of HDAC6 to induce ciliary disassembly. To explore this question, RPE1 cells were transfected with Flag-cortactin wild-type or mutants, including 9KQ (mutation of all nine of the repeat-region lysines to glutamines to mimic acetylation) and 9KR (mutation of all nine of the repeat-region lysines to glutamines to disrupt acetylation). 9KQ is known to be unable to interact with F-actin, and 9KR is known to preserve the ability to interact with F-actin^20^. We found that transfection of RPE1 cells with Flag-cortactin wild-type or 9KR significantly decreased the percentage of ciliated cells and the length of cilia, compared with transfection with Flag vector (Fig. 7A–C). By contrast, transfection with 9KQ slightly decreased ciliary length and did not obviously affect the percentage of ciliated cells (Fig. 7A–C).

To analyze whether the deacetylation of cortactin is necessary for HDAC6 to induce ciliary disassembly, RPE1 cells were transfected with GFP-HDAC6 together with Flag-cortactin wild-type, 9KQ, or 9KR. We found that cortactin wild-type and 9KR remarkably increased the ability of HDAC6 to induce ciliary disassembly, as indicated by the complete absence of cilia in the transfected cells (Fig. 7D–F). By contrast, 9KQ significantly counteracted the ciliary effect of HDAC6, as indicated by a striking increase in the percentage of ciliated cells and the length of cilia (Fig. 7D–F). These data suggest that the induction of ciliary disassembly by HDAC6 also requires the deacetylation of cortactin.

### Inhibition of actin polymerization abrogates HDAC6-mediated ciliary disassembly

**Figure 8:**
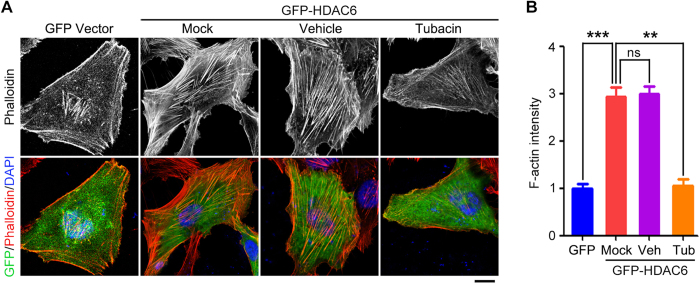
HDAC6 stimulates actin polymerization. (**A**) Immunofluorescence images of RPE-1 cells transfected with GFP vector or GFP-HDAC6, untreated or treated with tubacin (2 μM) or vehicle control, serum-starved for 24 hours, and stained with phalloidin and DAPI. Scale bar, 5 μm. (**B**) Experiments were performed as in A, and F-actin intensity was quantified. **P < 0.01, ***P < 0.001; ns, not significant. Error bars indicate SEM.

Since HDAC6 deacetylates cortactin and thereby increases the interaction of cortactin with F-actin^20^, we speculated that HDAC6 might promote actin polymerization. To test this possibility, RPE1 cells were transfected with GFP-HDAC6, and F-actin was stained with phalloidin. We found that transfection of GFP-HDAC6 significantly increased F-actin intensity, as compared with transfection with GFP vector (Fig. 8A,B). In addition, the HDAC6 inhibitor tubacin suppressed HDAC6-induced increase of F-actin staining (Fig. 8A,B). These data indicate that HDAC6 overexpression stimulates actin polymerization.

**Figure 9:**
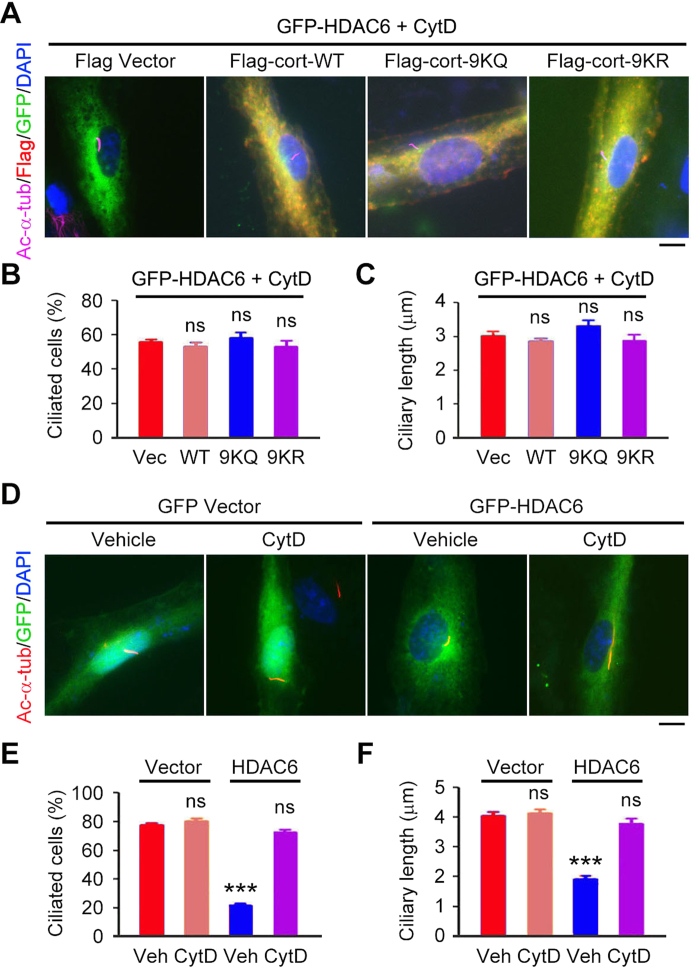
Inhibition of actin polymerization abrogates HDAC6-mediated ciliary disassembly. (**A**–**C**) Immunofluorescence images (**A**), percentage of ciliated cells (**B**), and ciliary length (**C**) in RPE1 cells transfected with GFP-HDAC6 and Flag vector or Flag-cortactin wild-type (WT), 9KQ, or 9KR, starved in serum-free medium with cytochalasin D (CytD, 5 mM) for 24 hours, and stained with acetylated α-tubulin and Flag antibodies and DAPI. Scale bar, 5 μm. (**D**–**F**) Immunofluorescence images (**D**), percentage of ciliated cells (**E**), and ciliary length (**F**) in RPE1 cells transfected with GFP vector or GFP-HDAC6, starved in serum-free medium with cytochalasin D (5 mM) or vehicle control for 24 hours, and stained with acetylated α-tubulin antibody and DAPI. Scale bar, 5 μm. ***P < 0.001; ns, not significant. Error bars indicate SEM.

Since branched F-actin is known to inhibit ciliogenesis^34^,^35^,^36^, we sought to investigate whether HDAC6/deacetylated cortactin-mediated increase of actin polymerization contributes to HDAC6-mediated ciliary disassembly. RPE1 cells were transfected with GFP-HDAC6 and Flag-cortactin wild-type, 9KQ, or 9KR, and then treated with cytochalasin D, a well-known inhibitor of actin polymerization. We found that cytochalasin D dramatically abolished the effect of cortactin on HDAC6-mediated ciliary disassembly (Fig. 9A–C).

To further investigate the role of actin polymerization in HDAC6-mediated ciliary disassembly, RPE1 cells were transfected with GFP vector or GFP-HDAC6 and then treated with cytochalasin D or vehicle control. We found that although cytochalasin D per se did not affect the percentage of ciliated cells or ciliary length, this drug could efficiently protect cells from HDAC6-induced ciliary disassembly (Fig. 9D–F). Taken together, these results suggest that actin polymerization contributes to the action of HDAC6 in triggering ciliary diassembly.

## Discussion

**Figure 10:**
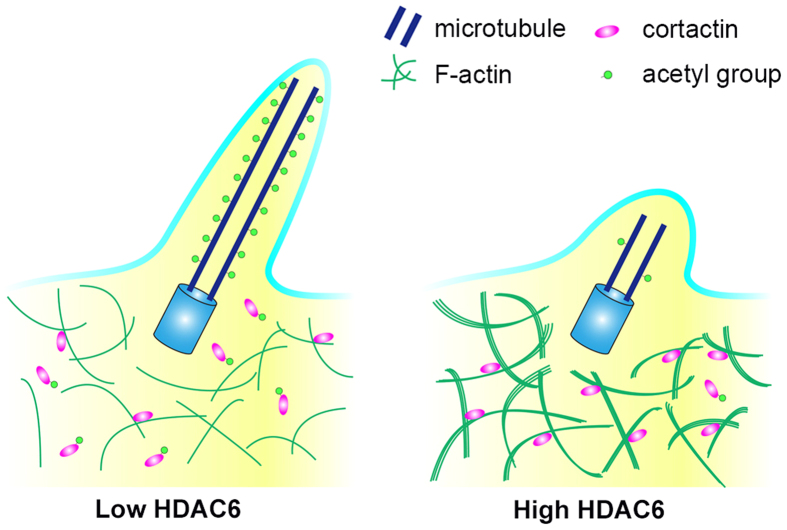
Molecular model for the role of HDAC6 in ciliary disassembly. HDAC6-mediated ciliary disassembly is dependent on the deacetylation of both α-tubulin and cortactin; deacetylation of α-tubulin reduces the stability of axoneme microtubules, whereas deacetylation of cortactin promotes its interaction with F-actin and accelerates actin polymerization, which in turn leads to ciliary resorption.

HDAC6 is a unique member of the HDAC family that mainly deacetylates non-histone substrates. A variety of proteins have been identified as HDAC6 substrates, including α-tubulin, cortactin, Hsp90, peroxiredoxin, and Tat^18^,^19^,^20^,^21^,^22^,^23^,^24^. By modulating the acetylation of these proteins, HDAC6 plays an important role in diverse cellular processes, such as cell motility and signaling^18^,^19^,^20^,^21^,^22^,^23^,^24^. Accumulating evidence has shown that HDAC6 is critically involved in ciliary disassembly^5^,^6^,^7^,^8^,^9^,^10^,^11^,^12^,^13^,^14^,^15^,^16^,^17^. However, the molecular mechanism of how HDAC6 functions in the control of cilia remains elusive, and it is unclear whether any known substrate of HDAC6 mediates its ciliary function. In this study, by a combination of multiple approaches, we demonstrate for the first time that deaetylation of α-tubulin and cortactin is required for HDAC6 to trigger ciliary disassembly; deacetylation of α-tubulin by HDAC6 reduces the stability of axoneme microtubules, whereas deacetylation of cortactin by HDAC6 promotes the interaction of cortactin with F-actin and accelerates actin polymerization, which in turn leads to ciliary resorption (Fig. 10).

Ciliary assembly and disassembly undergo dynamic regulation by diverse ciliary regulatory proteins including HDAC6. These proteins coordinate or counteract to maintain ciliary homeostasis. Our data show that overexpression of HDAC6 drives ciliary disassembly; however, knockdown of HDAC6 or inhibition of its activity has no obvious effect on cilia. It is possible that when HDAC6 is depleted or inhibited, other ciliary regulatory proteins substitutes its ciliary role. This may explain why depletion or inhibition of HDAC6 does not obviously affect cilia. When HDAC6 is overexpressed, its activity to trigger ciliary disassembly is significantly enhanced, switching the balance of ciliary homeostasis toward ciliary disassembly. This may explain why HDAC6 overexpression causes ciliary disassembly.

HDAC6 is known to regulate cell motility presumably by altering the acetylation status of α-tubulin and cortactin^19^,^20^,^37^,^38^,^39^. Cells overexpressing HDAC6 contain more deacetylated α-tubulin than control cells and are more motile^19^, supporting a role for HDAC6 in microtubule-dependent cell motility. Cilia, as microtubule-based organelles, contain more acetylated α-tubulin and stable microtubules in their axonemes, consistent with the role of HDAC6 in α-tubulin deacetylation and ciliary disasembly. In addition, HDAC6 decreases the level of cortactin acetylation, which enhances cortactin binding to F-actin and stimulates actin polymerization^20^, an important event that drives cell motility, manifesting the function of HDAC6 in actin-dependent cell motility. Ciliated cells harbor branched F-actin, similar to that present at the leading edge of migrating cells. However, different from its positive role in cell motility, branched F-actin inhibits ciliogenesis^34^,^35^,^36^. In this scenario, it is not difficult to understand why HDAC6-mediated deacetylation of cortactin contributes to ciliary disassembly while promoting cell motility.

It should be noted, however, that, although our data reveal α-tubulin and cortactin as important proteins mediating the ciliary role of HDAC6, it would not be surprising if other substrates of HDAC6 were identified in the future to fulfill this function. For example, myosin heavy chain 9 (MYH9) has recently been revealed as a novel HDAC6 substrate, and similar to cortactin, the actin-binding ability of MYH9 is regulated by its acetylation status^40^. It will be interesting to investigate in the future whether the deacetylation of MYH9 also contributes to the roles of HDAC6 in ciliary disassembly and cell motility. In addition, HDAC6-mediated autophagic turnover of ciliary proteins has recently been reported to be associated with ciliary dysfunction in the chronic obstructive pulmonary disease model^13^. Although our data show that overexpression or depletion of HDAC6 does not influence the level or localization of known ciliary regulators, we cannot completely exclude the possibility that HDAC6-induced ciliary disassembly might be partly attributed to an autophagy-dependent pathway.

Over the past decade, HDAC6 has become an attractive target for the development of drugs against a wide range of diseases. Unlike the inhibitors of many other HDACs, small-molecule inhibition of HDAC6 activity does not cause obvious toxicity. For example, the selective HDAC6 inhibitor tubastatin A has been investigated for the treatment of cancer, neurodegenerative disorders, and chronic obstructive pulmonary disease and displays no obvious side effect^13^,^41^,^42^,^43^. In the present study, we find that inhibition of HDAC6 activity by tubastatin A and tubacin does not affect the percentage of ciliated cells or ciliary length, but both drugs could protect cells from HDAC6-induced ciliary disassembly. Our data, together with previous findings that HDAC6 inhibitors could rescue various ciliary defects caused by the upregulation of HDAC6 expression or activity^9^,^10^,^11^,^12^,^13^,^16^,^41^, suggest that the therapeutic value of this class of agents for the treatment of HDAC6-associated ciliopathies merits further investigation.

## Methods

### Antibodies, chemicals, siRNAs, and plasmids

Antibodies against acetylated α-tubulin, γ-tubulin, Flag (Sigma-Aldrich), acetylated lysine, α-tubulin, Cep164, ninein (Santa Cruz Biotechnology), TMEM67, MKS1, IFT140, IFT88, CP110 (Proteintech), HDAC6 (abgent), and GFP (Roche) were purchased from the indicated sources. Rhodamine- and fluorescein-conjugated secondary antibodies were from Jackson ImmunoResearch Laboratories, and Alexa Fluor 405-conjugated secondary antibodies were from Invitrogen. Horseradish peroxidase-conjugated secondary antibodies were from Santa Cruz Biotechnology. Tubastatin A and tubacin were obtained from Santa Cruz Biotechnology. DAPI and tetramethylrhodamine-conjugated phalloidin were from Sigma-Aldrich. Control and HDAC6 siRNAs and plasmids expressing HA-HDAC6, GFP-HDAC6, and GFP-α-tubulin were described previously^38^,^44^, and various mutants were generated by PCR and site directed mutagenesis. The plasmid expressing mCherry-β-tubulin was constructed by PCR using the pmCherry-C1 vector (Clontech). Plasmids expressing Flag-cortactin wild-type, 9KQ, and 9KR plasmids were obtained from Edward Seto (H. Lee Moffitt Cancer Center & Research Institute, Tampa, USA).

### Cell culture and transfection

RPE1 cells were obtained from the American Type Culture Collection and grown in the DMEM/F12 medium supplemented with 10% FBS. To induce ciliary formation, cells were cultured in the serum-free medium for 24 hours. Plasmids were transfected into cells with the TurboFect reagent (Thermo Fisher Scientific) and siRNAs were transfected with the Lipofectamine RNAiMAX reagent (Invitrogen).

### Fluorescence microscopy

Cells were grown on glass coverslips and fixed with cold methanol for 5 minutes or with 4% paraformaldehyde for 20 minutes at room temperature followed by permeabilization with 0.5% Triton X-100 in phosphate-buffered saline (PBS) for 20 minutes. Cells were blocked with 2% bovine serum albumin in PBS for 30 minutes. Cells were incubated with primary antibodies for 2 hours at room temperature or overnight at 4 °C and then with secondary antibodies for 2 hours at room temperature. For F-actin staining, cells were stained with tetramethylrhodamine-conjugated phalloidin for 30 minutes as described^45^. Cells were then stained with DAPI for 5 minutes, mounted with anti-fade medium, and then examined with a TCS SP5 confocal microscope (Leica). The percentage of ciliated cells and the length of cilia were measured with the ImageJ software.

### Immunoprecipitation and Western blotting

For immunoprecipitation, cells were lysed in a buffer containing 50 mM Tris-HCl, 150 mM NaCl, 1 mM EDTA, 1.0% NP40, and 3% glycerol supplemented with the protease inhibitor cocktail (Roche). Cell lysates were incubated with primary antibody-coated agarose beads for 4 hours at room temperature or overnight at 4 °C. The beads were washed for 5 times in the lysis buffer, and the proteins were resolved by SDS-PAGE. For Western blotting, proteins were resolved by SDS-PAGE and transferred onto polyvinylidene difluoride membranes (Millipore). The membranes were blocked in Tris-buffered saline containing 0.2% Tween 20 and 5% non-fat milk and incubated with primary antibodies for 2 hours at room temperature or overnight at 4 °C and then with secondary antibodies for 45 minutes at room temperature. Immunoreactive signals were visualized with enhanced chemiluminescence detection reagent (Thermo Fisher Scientific).

### Statistics

Analysis of statistical significance was performed by the Student’s t-test using Microsoft Excel.

## Additional Information

**How to cite this article**: Ran, J. *et al.* Deacetylation of α-tubulin and cortactin is required for HDAC6 to trigger ciliary disassembly. *Sci. Rep.* **5**, 12917; doi: 10.1038/srep12917 (2015).

## Supplementary Material

Supplementary Figure S1, Figure S2
