# Supplementary material for: Deacetylation of α-tubulin and cortactin is required for HDAC6 to trigger ciliary disassembly
Source: Sci Rep. 2015 Aug 6;5:12917. doi: 10.1038/srep12917 (PMC4526867; doi:10.1038/srep12917)
Supplement: Supplementary Figure S1, Figure S2 [file srep12917-s1.pdf]

Supporting Information

Deacetylation of α-tubulin and cortactin is required for HDAC6 to trigger ciliary disassembly

Jie Ran, Yunfan Yang, Dengwen Li, Min Liu & Jun Zhou*

Figure S1. Knockdown of HDAC6 expression does not affect the expression or localization of ciliary regulators. (A) Western blot analysis of the indicated ciliary regulators, acetylated α-tubulin, α-tubulin, and HDAC6 in RPE1 cells transfected with control or HDAC6 siRNAs. (B) Immunofluorescence images of RPE1 cells transfected with control or HDAC6 siRNAs, serum-starved for 24 hours, and stained with antibodies against the indicated ciliary regulators and acetylated α-tubulin and DAPI. Scale bar, 5 μm.

Figure S2. Overexpression of α-tubulin decreases the percentage of ciliated cells and ciliary length. (A) Immunofluorescence images of RPE1 cells transfected with the indicated plasmids, serum-starved for 24 hours, and stained with acetylated α-tubulin antibody and DAPI. Scale bar, 5 μm. (B and C) Experiments were performed as in A, and the percentage of ciliated cells (B) and ciliary length (C) were quantified. ***$P < 0.001$; ns, not significant. Error bars indicate SEM.

Figure S1

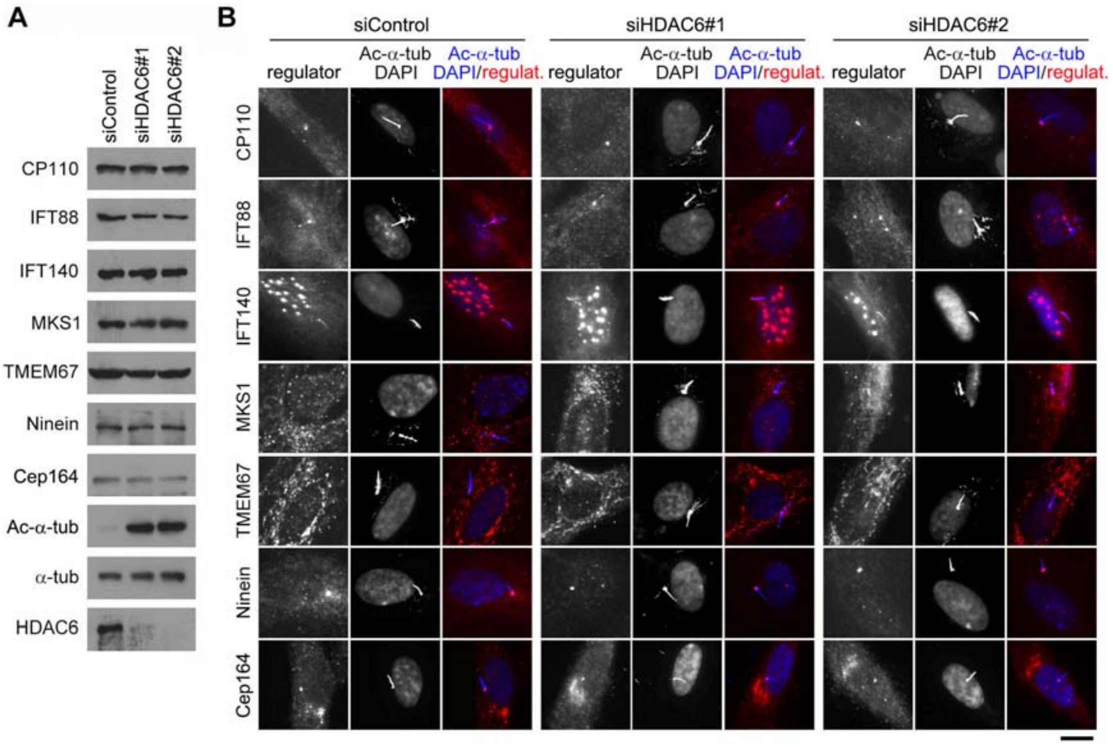
A
siControl
siHDAC6#1
siHDAC6#2
CP110
IFT88
IFT140
MKS1
TMEM67
Ninein
Cep164
Ac-α-tub
α-tub
HDAC6
B
siControl
siHDAC6#1
siHDAC6#2
regulator
Ac-α-tub DAPI
Ac-α-tub DAPI/regulat.
CP110
IFT88
IFT140
MKS1
TMEM67
Ninein
Cep164

Figure S2

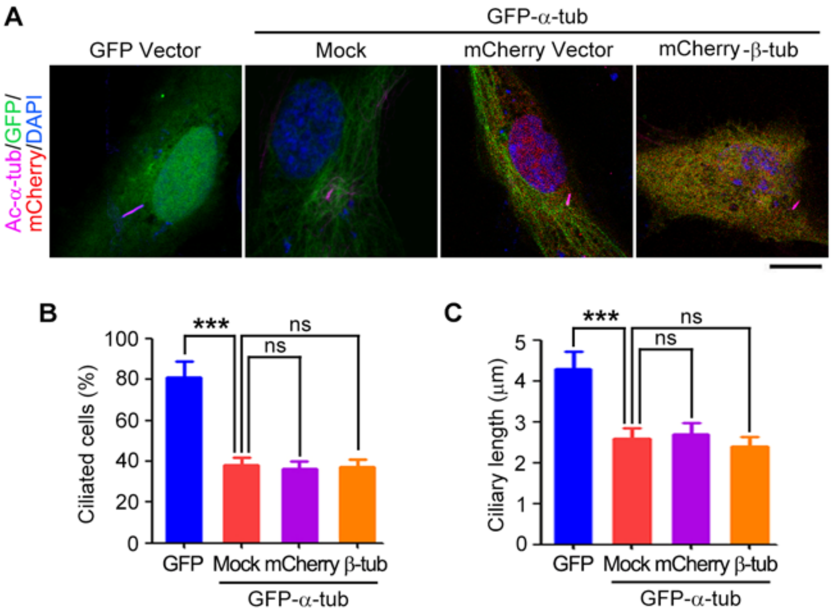
A
GFP-α-tub
GFP Vector
Mock
mCherry Vector
mCherry-β-tub
Ac-α-tub/GFP/
mCherry/DAPI
B
Ciliated cells (%)
100
80
60
40
20
0
***
ns
ns
GFP
Mock
mCherry
β-tub
GFP-α-tub
C
Ciliary length (μm)
5
4
3
2
1
0
***
ns
ns
GFP
Mock
mCherry
β-tub
GFP-α-tub
